# Stereotactic Body Radiation Therapy (SBRT) for lung cancer patients previously treated with conventional radiotherapy: a review

**DOI:** 10.1186/1748-717X-9-210

**Published:** 2014-09-19

**Authors:** Arya Amini, Norman Yeh, Laurie E Gaspar, Brian Kavanagh, Sana D Karam

**Affiliations:** Department of Radiation Oncology, The University of Colorado School of Medicine, 1665 Aurora Court, Room 1032, Aurora, CO 80045 USA

**Keywords:** Re-irradiation, NSCLC, SBRT, Toxicity

## Abstract

Lung cancer continues to be one of the most prevalent malignancies worldwide and is the leading cause of death in both men and women. Presently, local control rates are quite poor. Improvements in imaging and radiation treatment delivery systems however have provided radiation oncologists with new tools to better target these tumors. Stereotactic body radiation therapy (SBRT) is one such technique that has shown efficacy as upfront treatment for lung cancer. In addition, more recent studies have demonstrated some effectiveness in recurrent tumors in prior irradiated fields as well. This review summarizes seven recent studies of re-irradiation with SBRT in patients with thoracic recurrences treated previously with conventionally fractionated radiation therapy. Combined, 140 patients were included. The median initial thoracic radiation doses ranged from 50-87.5 Gy and median re-irradiation dose ranged from 40-80 Gy. Local control rates varied from 65-92%. Re-irradiation was well tolerated with few grade 4 and 5 complications (observed in one study). Currently, based on these published reports, re-irradiation with SBRT appears feasible for in-field thoracic recurrences, though caution must be taken in all cases of retreatment.

## Introduction

There are approximately 228,000 new cases of lung cancer diagnosed in the United States each year and approximately 70% of patients with lung cancer receive external beam radiation treatment (EBRT) as one component of their treatment [1]. Despite advancements in imaging, staging and treatment with surgery, radiation, and chemotherapy, rates of locoregional recurrence in lung cancer continue to be high. Studies have cited thoracic recurrences or new metachronous primary lung tumor in the range of 4-10% [2, 3]. Other studies using bronchoscopy to evaluate response after treatment found significantly worse outcomes, with less than 20% of later stage non-small cell lung cancer patients experiencing a complete resolution of their disease 3 months after standard course fractionation (2 Gy) [4]. As systemic treatment continues to improve, local disease control has become increasingly important. The treatment of thoracic recurrences, in particular those within prior radiation fields, pose a significant challenge for radiation oncologists. Stereotactic body radiation therapy (SBRT) has become a more commonly used modality to treat both primary and recurrent disease and has significantly impacted the local control rates in lung cancer.

SBRT was derived from intracranial stereotactic radiosurgery (SRS), which was first presented in 1951 [5]. In 2001, SRS was approved by the US Food and Drug Administration to treat areas throughout the body and the first SBRT delivery system was established. Immobilization devices and improved real-time imaging have allowed clinicians to administer high ablative doses to accurately target the tumor. The effectiveness of SBRT arises from the cumulative biologically effective dose (BED) it can achieve while maintaining a sharp dose gradient fall off outside the target, preventing dose to critical structures. Reaching a high BED has been shown to improve overall survival and local tumor control rates in many tumors [6]. Martel and colleagues published a dose-escalation study in patients with inoperable non-small cell lung cancer (NSCLC), achieving doses as high as 103 Gy in 2 Gy fractions [7]. The study demonstrated a sigmoid-shape dose response between dose and recurrence free survival, suggesting doses up to 84 Gy are needed to achieve greater progression-free survival. A review of 13 randomized controlled trials for palliative thoracic radiotherapy found improvements in symptom control and survival at one year with 35 Gy_10_ BED schedules [8]. Onishi et al [9] demonstrated a local control rate of 92% for those receiving SBRT with a BED > 100 Gy compared to 74% with a BED < 100 Gy. In addition, higher dose per fraction treatments given over a shortened timed frame in comparison to conventional fractionation may reduce the rate of repopulation in tumor cells [10]. More comformal delivery also leads to smaller treatment field margins which in turn relates to lower toxicity and is especially important in the setting of re-irradiation.

For recurrent disease in the thorax, especially within the previous radiation field, salvage surgery is typically avoided. Therefore most institutions favor chemotherapy. Unfortunately the current response rates for second and third line chemotherapy remain quite poor, leaving fairly few options for these patients [11]. The use of re-irradiation has been studied in several regions of the body, most notably in the head and neck. Overall, retreatment in these studies was well tolerated and local control rates were acceptable [12, 13]. For example, a phase I SBRT, dose-escalation, re-irradiation study for patients with relapsed head and neck squamous cell cancer demonstrated tolerable side effects in those treated up to 44 Gy in 5 fractions given over 2 weeks [12]. Treatment response was observed in up to 76% of patients in the study, with a median disease free progression of 4 months. There were no grade 3 or 4 toxicities. Extrapolating from this data and the manuscripts reviewed here, SBRT is suggested to be an advantageous approach for recurrent NSCLC after prior conventional radiation.

### SBRT in recurrent thoracic tumors

#### SBRT for lung tumors

SBRT for primary treatment of early stage lung cancer has been well established with multiple prospective trials [14]. Local control rates for early stage lung cancer using SBRT are greater than 90% [15–17]. The initial phase I study assessing dose escalation using SBRT for inoperable lung cancer used starting doses of 24 Gy in 3 fractions [15]. The maximum-tolerated dose was never achieved for T1 tumors while T2 tumors reached 60-66 Gy in 3 fractions with tolerable toxicities. Dose limiting toxicities for larger tumors (5-7 cm) included bronchial injury, pneumonia, and pericardial effusion. The phase II study used a total dose of 60-66 Gy in 3 fractions [18]. Three-year cancer-specific survival in this study was 81.7%. Grade 3 to 5 toxicity occurred more commonly in central tumors (27.3% versus 10.4%) [19]. While there are a number of studies describing upfront SBRT for NSCLC, there is little data describing the use of SBRT in the re-irradiation setting for NSCLC.

The following section will present fairly recent data on SBRT for lung cancer patients previously treated with conventional fractionation. There are a number of limitations addressed in these studies. Overall patient numbers are few and follow up is short. Patient selection and population heterogeneity in those retreated provides some difficulty when comparing results of these studies conducted at the various institutions discussed. Patients included in the study had re-irradiation at any location in the thorax, not necessarily overlapping the initial field of treatment and often times in a separate ipsilateral or contralateral lobe. Toxicity rates and outcomes would likely vary substantially depending on the proportion of patients retreated with true overlapping fields. In addition, the radiation delivery technique and dosing used is quite variable across these studies. Lastly, there is selection bias in those receiving re-irradiation as they are usually better equipped to tolerate re-irradiation and usually have limited systemic progression compared to those who are not candidates [20].

#### Summary of re-irradiation studies

SBRT for thoracic re-irradiation has only recently been published (Table 1). These studies are small in nature. Several as will be mentioned include both re-irradiation in and outside the original treatment field. The first is a retrospective study conducted at MD Anderson analyzing outcomes in retreated lung cancer patients with SBRT [21]. The study included all thoracic re-irradiation cases (both in and outside the original treatment field). In field re-irradiation including any overlap within the prior high dose region defined as >30 Gy. The most common re-irradiation regimen was 50 Gy in 4 fractions (72% of patients). Local control rates were 92%. Three out of the 22 intrathoracic failures occurred within the SBRT field and of those, 2 had received suboptimal dosing and coverage. Patients treated for out-of-field relapses had improved progression free survival time as compared to patients with in-field relapses. Median overall survival at 2 years was 59%. Toxicities noted in the study included grade 3 pneumonitis (28%), grade 3 esophagitis (8%), grade 3 skin changes (6%), and grade 3 cough (3%). Grade 3 pneumonitis correlated with retreatment of out-of-field relapse (p = 0.03). No grade 3 pneumonitis was observed with in-field retreatment. There were no grade 4 or 5 toxicities reported.

**Table 1:** **SBRT Thoracic Re-irradiation Studies**

Study	Coon et al [24]	Kelly et al [21]	Trakul et al [22]	Reyngold et al [23]	Ester et al [25]	Trovo et al [27]	Seung et al [26]
Number of Patients	12	36	15	39	13	17	8
First Radiation Dose	n/a	61.5 Gy (range 30-79.2)	Median BED 87.5 Gy (range 60-112.5)	61.0 Gy (30-80)	61.2 Gy (median)	50-60 Gy	50-68 Gy
Time Between Treatments (median)	n/a	22.0 mo (range 0-92)	16 mo (range 5-80)	37.0 mo (range 1-180)	19.7 mo (range 4.7-84.7)	18 mo (range 1-60)	367.0 mo (range 8-57)
Re-irradiation Dose	60 Gy	50 Gy (72%), 40 Gy (17%), Other (11%)	median BED 80 Gy (60-112.5)	median BED 70.4 Gy (range 42.6-180)	9-10 Gy x5	30 Gy (5-6 fx)	12 Gy x4, 10 Gy x5, 8 Gy x5, 20 Gy x3
Target Size (range)	median GTV 14.3 cc	tumor (median) 1.7 cm (range 0.6-3.8)	14.2 ml (range 2-57.7)	median GTV 19.0 cc (0.7-227)	n/a	n/a	n/a
Follow up (median)	12 mo	15 mo (range 4-45)	15 mo (range 4-65)	12.6 mo (range 1.3-47.5)	11.4 mo (0.9-38.3)	18 mo (range 4-57)	18 mo (range 11-20)
Local Control	92%	92%	65.5%	77% (1-year LPFS)	92%	86%	86%
Overall Survival	67% (1-year)	59% (2-years)	80% (1-year)	22.0 months (MS)	n/a	59% (1-year)	n/a
Toxicity	no G3	G3 pneumonitis (28%), G3 esophagitis (8%), G3 skin (6%), G3 cough (3%), No G4/5 toxicities	chest wall pain (6.7%), esophagitis (0.9%), no G2 or higher pneumonitis, no G4 or higher toxicities	G2 pulmonary (18%), G3 pulmonary (5%), G2/3 chest wall pain (18%), G2/3 fatigue (15%), G2-4 skin toxicity (5%), no G5 toxicities	G2 pulmonary (7.7%), G3 pulmonary (7.7%), no G4 or G5	G3 pneumonitis (23%), G5 pneumonitis (6%), G5 hemoptysis (6%)	G1 cough (13%), G1 pain (13%), G2 dyspnea (100%)

n/a: not available; mo: months; G: grade.

Stanford University recently published their SBRT re-irradiation experience as well [22]. The study included 15 patients treated with SBRT to in-field recurrences alone. This included patients initially treated with either conventional fractionation (n = 11) or SBRT (n = 4). They compared this cohort to those patients at their institution who received primary SBRT as their initial treatment. Median biologic effective dose (BED) for re-irradiation was 80 Gy. When comparing outcomes of SBRT for recurrent in-field tumors compared to up front SBRT for primary/initial treatment, 12-month local control rates were 65.5% compared to 92.1% respectively. Kaplan-Meier estimates demonstrated those with a shorter interval between treatments (≤16 months) had lower rates of local control (46.5% vs. 87.5%, p = 0.042). Re-irradiation was well tolerated in all cases, including grade 2 or higher pneumonitis (11.6%), chest wall pain (6.7%) and esophagitis (0.9%). One individual developed ipsilateral vocal cord paralysis which may have been treatment related as the treated site was located adjacent to the aortic notch.

Reyngold et al [23] also recently published the results of 39 patients with prior intra-thoracic conventional radiation who underwent SBRT for either a new primary, recurrent (in or out-of-field) or metastatic lung tumor at Memorial Sloan Kettering Cancer Center. Of the 39 patients retreated, 22 (56%) individuals had overlap with prior radiation fields. Multiple SBRT regimens were included: 60 Gy, 50 Gy and 48 Gy in 3 fractions, 40-45 Gy in 5 fractions, 20-22 Gy in 1 fraction, 32-35 Gy in 4-5 fractions, and 27.5-30 Gy in 5 fractions. The local progression free survival (LPFS) rates at 1- and 2-years for the entire cohort were 77% and 64% respectively. Toxicities included grade 2 or 3 pulmonary (dyspnea, hypoxia, cough, pneumonitis) (23%), chest wall pain (18%), fatigue (15%), and skin/soft tissue breakdown (5%). There were no reported grade 5 toxicities. SBRT doses with a BED_10_ ≥ 100 Gy vs. < 100 Gy and overlap with the prior radiation field did not correlate with pulmonary toxicity. Factors associated with improvement in LPFS were no overlap with the prior radiation field (p = 0.04), BED_10_ ≥ 100 Gy (p = 0.04), time interval greater than 36 months between treatments (p = 0.05), PTV < 75 cm^3^ (p = 0.03), and KPS ≥ 80 (p = 0.03).

Coon et al [24] published their data from the University of Pittsburgh Cancer Center. The study included 12 patients with residual/recurrent lung cancer after previous treatment. Patients were treated to a dose of 60 Gy in 3 fractions, prescribed to the 80% isodose line. After a median follow up of 12 months, local control rates were 92% in patients with recurrent lung cancer. Treatment was well tolerated with one grade 2 radiation pneumonitis and another patient with exacerbation of chronic obstructive pulmonary disease, which reportedly was treatment and/or tumor related with no grade assigned. The University of Minnesota recently presented their re-irradiation experience as well [25]. Ten patients included had initially received conventional fractionation followed by re-irradiation with SBRT. Local control rates were 92% and pulmonary toxicities included one grade 2, one grade 3, and no grade 4 or 5 complications. Seung et al [26] looked at their institutional experience as well, including 8 patients retreated with SBRT after definitive radiation therapy and found local control rates of 86%. Pulmonary toxicities include one grade 1 cough, one grade 1 pain, eight grade 2 dyspnea with no grade 3 or greater side effects.

Most recently, a group in Italy published their retrospective data on 17 patients with in-field centrally located NSCLC who received re-irradiation with SBRT [27]. Centrally located tumors were defined as being located 2 cm in all directions around the proximal bronchial tree. All patients included were treated with prior conventional radiation therapy (50-60 Gy in 20-30 fractions) and only in-field recurrences were included. The SBRT dose given was 30 Gy in 5 or 6 treatments with cumulative doses ranging from 87-100 Gy. With a follow up of 18 months, local control rates were 86% with 1 and 2 year overall survival rates of 59% and 29% respectively. The significance of this study was the reported grade 5 toxicities, which included one patient who died of hemoptysis 2 months after finishing treatment and another patient who died from pneumonitis 4 months after treatment. Patients in the study who developed grade 3-5 pneumonitis had a higher heart maximum dose (Dmax), a minimum dose to at least 5% of the heart volume (D5) mean of 10 Gy, and a minimum dose to at least 10% of the heart volume (D10) mean of 3 Gy (p < 0.05).

#### Toxicities

As there is relatively little data published on re-irradiation using SBRT, the side effect profile is still not fully characterized. Acute toxicities from SBRT may be as common as 40% and include fatigue, skin erythema, hematologic suppression and cough; late toxicities are less common and included pneumonitis, scarring, worsening pulmonary function, chest wall pain, rib fractures, esophageal injury and brachial plexopathy [28]. Pneumonitis appears to be the most common side effect from retreatment, up to 40% in some cases [21]. Liu et al [29] analyzed rates of pneumonitis in those receiving conventional fractionation followed by re-irradiation with SBRT for recurrent disease. Rates of severe pneumonitis were common (20.8%), with predictive factors including performance status, forced expiratory volume in one second (FEV1), initial planned tumor volume (PTV) including bilateral mediastinum, and those receiving doses ≥ 20 Gy (V_20_) to 30% or more of the composite volume. In general, long term side effects in these patients are more difficult to assess given the shortened median survival in patients with recurrent lung tumors. Evans et al [30] found grade 5 aortic toxicities as high as 6% in patients with composite doses ≥ 120 Gy to the aorta, making note that both severe and fatal toxicities can and do exist. As described earlier, Trovo et al [27] unfortunately experienced two treatment-related deaths in their re-irradiation study as these were centrally located recurrences. With fairly high rates of toxicities seen in multiple thoracic re-irradiation studies as described above, physicians must remain vigilant to normal tissue structure doses, especially in cases of re-irradiation near critical structures.

## Discussion

For many patients, especially in the recurrent setting, surgery may not be a feasible option. Other local therapy approaches such as radiofrequency ablation (RFA) have also been studied for NSCLC but local control rates overall are lower when compared to SBRT [31]. Therefore, when administered safely, as demonstrated in the studies discussed here, SBRT is a viable option for in-field or out-of-field lung recurrences. Evidence shows high rates of local control for early-stage NSCLC but there is limited data on re-irradiation using SBRT. As discussed earlier, present studies include a heterogeneous population of patients initially treated with conventional fractionation followed be both in-field or out-of-field recurrences treated with SBRT. Other than Trovo et al [27] which included centrally located recurrences, other re-irradiation studies did not define the location of the recurrences or comment on correlation between toxicity and central versus peripheral re-irradiation. Longer follow up is needed to further evaluate late toxicity which is often a concern with high dose fractionation.

However, based on the data reviewed here, use of SBRT for lung re-irradiation, when accounting for normal-tissue tolerance and location in relation to critical structures, may be a reasonable salvage option for patients. With improved imaging and radiation techniques, treatment modalities such as SBRT will continue to grow and be more commonly utilized throughout the country. Much of this stems from better targeting with the use of state of the art image guidance. The introduction of positron emission tomography (PET) and computerized tomography (CT) for example has come to play large role in diagnosing, staging, and providing radiation oncologists with the ability to further optimize treatment plans, treating smaller volumes with more precise targeting [32–35]. Results from the ACRIN 6668/RTOG 0235 trial recently published found post-treatment tumor standardized uptake value (SUV) to be predictive of outcomes and survival [36]. In addition, PET scans have allowed for earlier detection of small, solitary tumors and will continue to play a major part in identifying early recurrences that may be amenable to re-irradiation with SBRT [37]. Incorporation of PET for planning purposes also provides better delineation of the tumor itself, thus reducing volume and allowing radiation planners to meet normal-tissue toxicity parameters, especially in the setting of re-irradiation. For example, PET can help delineate primary tumors from atelectasis, thus decreasing final target volumes [38]. Use of PET therefore could play a role in not only identifying early stage lung tumors, but also assisting in better target delineation during treatment planning. The additional challenge of treatment planning is accounting for tumor motion. There are several techniques to evaluate tumor motion and assist in treatment planning, including four-dimensional (4D) CT, respiratory gating and active breathing control [39–41]. Three-dimensional (3D) assessment of a tumor can be obtained by repeating CT scans acquired asynchronously based on breathing patterns or CT scans can be repeated at different states of a breathing cycle to define tumor motion [10, 42]. These techniques help to provide more accurate dose delivery, allowing radiation oncologists to deliver higher doses with greater conformality and accuracy while minimizing toxicity [43, 44].

Despite improvements in radiation delivery techniques and biologic advances in chemotherapy, locoregional control continues to remain a challenge in thoracic tumors, with failure rates as high as 85% [20, 45]. Furthermore, retreatment of locoregional recurrences can be quite difficult as therapeutic options are still relatively limited. Options other than SBRT include conventional or hypofractionated re-irradiation. A recent review included eleven studies reporting outcomes for thoracic re-irradiation of locally recurrent non-small cell lung cancers (NSCLC) with conventional or hypofractionated doses [46]. The studies included in this review rarely used intensity modulated radiotherapy (IMRT) and none included retreatment with SBRT. The goals of re-irradiation for the majority of these patients were palliative. Symptom control ranged broadly from 33% - 100%, with significant improvements observed in rates of hemoptysis, cough, chest pain and dyspnea. Median survival times following re-irradiation ranged from 5 to 14 months while one and two-year survival rates ranged from 8.7 to 59% respectively. The review also demonstrated a trend for longer survival times with higher doses of radiation and found that overall high grade toxicity was relatively low (3-5%). Toxicities were mostly grade 1-3, including pneumonitis, rib fractures, skin toxicity and esophagitis. One treatment-related death secondary to pneumonitis versus tumor progression was reported.

Considering re-irradiation with more conventional fractionation over five to six weeks with chemotherapy may be an option but can be difficult for some patients to tolerate as many lung cancer patients also present with a host of co-morbid illnesses, contributing to their poor performance status. For elderly patients with poor prognostic factors including weight loss, Karnofsky performance status (KPS) scores < 70 and additional health comorbidities, chemotherapy and more conventional dosing with re-irradiation given over 4-6 weeks may be difficult to tolerate [47]. Several studies have demonstrated the significant rates of increased toxicity and overall worse survival in elderly patients with multiple co-morbidities treated for lung cancer [48, 49]. This population in particular, may benefit substantially from SBRT, as it can be delivered in a much shorter treatment course making it more convenient and tolerable for the patient. Lastly, stereotactic radiotherapy provides a shortened treatment interval, which can be effective in local tumor control while minimizing dose fall off to nearby critical structures. Radiation oncologists however must continue to remain cognizant as toxicities can be high and fatal as was demonstrated in the recent Italian study described earlier [27].

There are some limitations in the literature reviewed in this manuscript. All studies include a short median follow up, ranging from 11-18 months. This is not only pertinent when analyzing local control rates but also late toxicities including lung fibrosis which more commonly occurs 1-2 years after the completion of radiation [50]. Radiation treatment changes on lung parenchyma often are not visible until 2-3 years following treatment [51, 52]. In addition, SBRT is more feasible for smaller volume recurrences given normal tissue constraints, especially in the setting of re-irradiation. As described earlier, the described literature has small patient numbers and includes a heterogeneous population with some patients included with re-irradiation within the previous field or outside it making interpretation of some of these studies very difficult. Description of the initial treatment is also lacking so concluding dose per fraction (conventional vs. hypofractionation) is not clearly defined. Lastly, several important factors are not described in the manuscripts presented, including the number of central tumors retreated, inclusion of both BED and physical dose in the papers, and correlation of severe toxicity and cumulative re-irradiation dose. Future studies will need to analyze re-irradiation dose tolerances of centrally located recurrences as toxicity rates are significantly higher for these patients as demonstrated by Trovo et al [27].

While there is substantial data and guidelines available for normal tissue tolerance in upfront radiation treatment, there are few guidelines available for radiation oncologists to follow for re-irradiation using SBRT. As discussed earlier, SBRT for upfront treatment of early stage NSCLC have high local control rates [19]. The currently limited amount of retrospective data available and reviewed in this manuscript for re-irradiation using SBRT also demonstrates fairly high rates of local control and tolerable toxicity outcomes with only a few cases of grade 4 side effects and grade 5 (observed in one study). Overall, re-irradiation for recurrent or persistent lung cancers using SBRT is feasible and should be offered to patients, taking into account performance status, individual comorbidities and the experience of the clinician in delivering treatment using these sophisticated techniques. Other local treatment options such as surgery, RFA, and systemic agents should be discussed.

## Conclusions

SBRT is a relatively effective, convenient and tolerable retreatment option for recurrent thoracic tumors treated prior with conventional fractionation. Overall toxicities, while prevalent, are mostly tolerated by patients. Future studies incorporating systemic biologic agents will need to be conducted to evaluate efficacy and toxicity of combined modality treatment. At the present time, SBRT is a reasonable option for re-irradiation in previously treated lung tumors. Care must be taken in the cumulative dose to nearby critical structures and patient comorbidities, present disease burden, time from initial treatment and overall prognosis should be assessed prior to proceeding with re-irradiation.

### Search strategy and selection criteria

We searched PubMed with the following keywords used in various combinations: “lung cancer”, “carcinoma”, “local failure”, “local recurrence”, “stereotactic body radiation therapy (SBRT)”, “staging”, “metastasis”, “treatment”, “management”, “chemotherapy”, “radiation therapy”, “outcomes”, “retreatment”, “survival”, and “recurrent disease”. The search was limited to articles published in peer-reviewed journals published from 1980 through December 2013. Articles were also identified through searches of the authors’ own files. Only papers published in English were reviewed. The final reference list was generated on the basis of relevance to the scope of this Review.
